# Clinicians’ Prediction of Survival Is Most Useful for Palliative Care Referral

**DOI:** 10.1089/pmr.2024.0013

**Published:** 2024-08-21

**Authors:** Eun Hee Jung, Yusuke Hiratsuka, Sang-Yeon Suh, Seok-Joon Yoon, Beodeul Kang, Si Won Lee, Koung Jin Suh, Ji-Won Kim, Se Hyun Kim, Jin Won Kim, Keun-Wook Lee, Yu Jung Kim

**Affiliations:** ^1^ Division of Hematology and Medical Oncology, Department of Internal Medicine, Seoul National University Bundang Hospital, Seoul National University College of Medicine, Seongnam, South Korea.; ^2^ Department of Palliative Medicine, Takeda General Hospital, Aizu Wakamatsu, Japan.; ^3^ Department of Palliative Medicine, Tohoku University Graduate School of Medicine, Sendai, Japan.; ^4^ Department of Family Medicine, Dongguk University Ilsan Hospital, Goyang-si, South Korea.; ^5^ Department of Medicine, College of Medicine, Dongguk University, Seoul, South Korea.; ^6^ Department of Family Medicine, Chungnam National University Hospital, Daejeon, South Korea.; ^7^ Division of Medical Oncology, Bundang Medical Center, CHA University, Seongnam-si, South Korea.; ^8^ Palliative Care Center, Yonsei Cancer Center, Yonsei University Health System, Seoul, South Korea.; ^9^ Division of Medical Oncology, Yonsei Cancer Center, Yonsei University Health System, Seoul, South Korea.

**Keywords:** advanced cancer, palliative care, prediction, survival

## Abstract

**Background:**

Timely palliative transition in patients with advanced cancer is essential for their improved quality of life and overall survival (OS). Most prognostic models have been developed focusing on weeks’ survival. The current study aimed to compare the accuracies of several indicators, such as the Karnofsky Performance Scale (KPS), Clinicians’ Prediction of Survival (CPS), and Edmonton Symptom Assessment System (ESAS), for predicting the survival of patients.

**Methods:**

Two hundred patients were enrolled at a single tertiary cancer center in South Korea between 2016 and 2019. We compared the discrimination of CPS versus KPS and ESAS total scores using the area under the receiver operating characteristic curve (AUROC) in 3-month and 6-month survival predictions.

**Results:**

The median age of patients was 66.0 years, and 128 (64%) were male. Two-thirds (66%) of the patients had an Eastern Cooperative Oncology Group performance status of 0 or 1, and 55.5% had a KPS of 80% or higher. The values of AUROC of CPS, KPS, and ESAS total score in 3-month survival prediction were 0.80 (95% confidence interval [CI]: 0.73–0.88), 0.71 (95% CI: 0.62–0.79), and 0.71 (95% CI: 0.62–0.81), respectively, whereas those in 6-month survival were 0.82 (95% CI: 0.76–0.88), 0.70 (95% CI: 0.63–0.78), and 0.63 (95% CI: 0.55–0.71), respectively.

**Conclusion:**

CPS showed the highest accuracy in predicting 3- and 6-month survival, whereas KPS had an acceptable accuracy. Experienced clinicians can rely on CPS to predict survival in months. We recommend the use of KPS with CPS to assist inexperienced clinicians.

## Introduction

Patients with advanced cancer experience various cancer-related symptoms that reduce their quality of life (QoL). Referral to timely palliative care is important for alleviating symptom burden and improving the QoL and mood of patients with advanced cancer.^1,2^ Recent studies and guidelines have recommended the initiation of early palliative care concurrently with palliative chemotherapy, specifically.^1–4^

Despite the positive outcomes of palliative care, its introduction has sometimes been too late to draw meaningful benefits on the disease trajectory.^5–7^ However, predicting survival remains challenging. A previous study reported a gap between prognostication and real data.^8^ Researchers have developed various prognostic and predictive models, over the past decades, to prepare for timely palliative transition and advanced care planning (ACP). Karnofsky Performance Scale (KPS), the performance status scale, has been reported to predict survival as a component of the palliative prognostic score.^9^ Clinicians’ prediction of survival (CPS) is a subjective prognostic marker that is known to be more accurate in patients with shorter life expectancies.^10–14^ Previous studies had reported that symptom burden of patients and health-related QoL are factors related to survival prediction.^15–18^ The Edmonton Symptom Assessment System (ESAS) score was a widely used tool for assessing patient-reported outcomes to evaluate the symptom burden in patients with advanced cancer. The ESAS score has also been reported to predict survival in patients with cancer.^19–21^ Most prognostic tools have been used to predict survival over weeks in patients with advanced cancer, and the Palliative Prognostic Index, a widely used index, has shown high specificity and sensitivity in predicting survival rates of <3 or 6 weeks.^22^ However, very few tools have been developed till date for predicting survival over months.^23^ A few studies have reported that the KPS and patient symptom burden are associated with the prediction of survival over months.^24,25^

Numerous attempts have been made to investigate survival prediction models by comparing them to various assessment tools.^26–28^ The decision to initiate palliative care is even more complex with the advent of an era that has shown changes in treatment schemes caused by the development of immunotherapy and targeted drugs. The current study aimed to identify and compare the accuracies of various prognostic models in patients with advanced cancer, who were expected to survive for more than a month, for an appropriate transition to palliative care.

## Materials and Methods

### Study design and subjects

Patients with advanced cancer, who were treated at a tertiary cancer center located in a metropolitan area of South Korea between March 2016 and January 2019, were enrolled in the study. The patients were considered eligible based on the following criteria: (1) age >18 years, (2) diagnosis of advanced cancer, and (3) expected survival of <1 year, as predicted by the oncologist in charge of the patients. In particular, we focused on patients whose survival was predicted by month–survival time in actual practice. We excluded patients with (1) hematologic malignancy, (2) inability to communicate with medical staff, and (3) prediction of survival of <1 month, which was evaluated by physicians using CPS. Patients with hematological malignancies were excluded because they underwent different treatment modalities and disease courses. For instance, they can be treated with curative intent through stem cell transplantation or chimeric antigen receptor T cell therapy, even in a refractory/relapse setting. Advanced cancer was defined as a progressive, locally advanced disease or metastatic or recurrent disease that could not be treated with curative intent. Based on previous studies, we selected the survival prediction times as 3 and 6 months, as 3-month survival is the minimal referral time for palliative transition, and a timely transition to palliative care is referred to as a median survival of <1 year.^29,30^ Written informed consent was obtained from each patient before enrollment. The study protocol was approved by the Institutional Review Board (IRB) of the study site (IRB number: B-1601/332-302).

### Data collection

We obtained demographic data and clinical characteristics, including the primary tumor site and type of chemotherapy, from electronic medical records. The clinical research nurse interviewed the patients face-to-face after enrollment. Performance status of patients was evaluated using the KPS. Cancer-related symptom burden was assessed using the Korean Edmonton Symptom Assessment System (K-ESAS), which has been validated in Korean patients with cancer.^31^ The sum of each variable of ESAS was used in the analysis as the total score of ESAS. The physicians were asked to estimate the CPS using the temporal question: “How many weeks do you think this patient will have?” The CPS was categorized by weeks. We also collected data on the characteristics of the participating physicians.

### Statistical analysis

We calculated frequencies and proportions for categorical variables, and median and interquartile range or mean and standard deviation for quantitative variables. We evaluated the performance of these variables in terms of discrimination and calibration. The discriminatory ability of each tool, for prediction, was measured using the area under the receiver operating characteristic curve (AUROC). The AUROC is a statistical measure used to evaluate the discriminatory power of a diagnostic test that displays the sensitivity and specificity of various cutoff values for predicting an outcome at some point in the future.^32^ The AUROC value ranges from 0.5 to 1, with 0.5 indicating no discrimination and 1 indicating perfect discrimination. Briefly, the higher the AUROC value, the higher the probability of the given test correctly classifying a binary outcome. The cutoff value of each variable in predicting survival was as follows: (1) As for ESAS total score, cutoff was the median; 2) regarding KPS score, it was <60 according to the distribution of our data; and 3) CPS was categorized into being consistent with the survival prediction target of 3 and 6 months, respectively. Performance of each prognostic indicator for 3-month and 6-month survival was assessed using sensitivity, specificity, positive predictive value (PPV), negative predictive value (NPV), and overall accuracy (OA). The confidence intervals (CIs) were calculated at a 95% confidence level. Finally, we used a calibration plot (observed vs. predicted graph) and a logistic regression model to assess the calibration. The CPS, KPS, and ESAS total scores were considered as continuous variables in the calibration assessment. All data were analyzed using JMP (version 14.0; SAS Institute, Cary, NC, USA) and IBM SPSS Statistics for Windows (version 26.0; IBM Corp., Armonk, NY, USA).

## Results

### Patient characteristics

During the study period, 200 patients with advanced cancer, who were treated at the outpatient medical oncology clinic of a tertiary university hospital, were enrolled. The median age of the patients was 66.0 years (range, 32–85 years) and 128 (64%) were male. Lungs were the most common sites of primary malignancy (*n* = 67, 33.5%), followed by the genitourinary tract (*n* = 29, 14.5%), colorectum (*n* = 28, 14.0%), and stomach (*n* = 20, 10.0%). Two-thirds (66%) of the patients had an Eastern Cooperative Oncology Group performance status of 0 or 1, and 55.5% had a KPS of 80% or higher. A total of 133 patients (65.5%) were receiving chemotherapy at the time of enrollment (Supplementary Table 1). The median overall survival was 7.6 months (range, 6.52–8.68). Baseline characteristics of the patients and their ESAS scores are shown in Table 1.

**Table 1.** Baseline Characteristics of Patients

Characteristics	*n* = 200 (%)
Age, years (median, range)	66.0 (32.0–85.0)
Sex	
Male	128 (64.0)
Female	72 (36.0)
Primary cancer site	
Lung	67 (33.5)
Stomach	20 (10.0)
Colon/Rectal	28 (14.0)
Ovary/Cervical	4 (2.0)
Liver/Biliary tract	4 (2.0)
Pancreas	4 (2.0)
Esophagus	5 (2.5)
Head/Neck	4 (2.0)
Soft tissue	6 (3.0)
Kidney/Bladder	29 (14.5)
Breast	18 (9.0)
Others	11 (5.5)
Metastasis site	
Lung	104 (52.0)
Liver	74 (37.0)
Brain	39 (19.5)
Bone	66 (33.0)
Lymph nodes	114 (57.0)
Others	77 (38.5)
Undergoing chemotherapy (Yes)	131 (65.5)
KPS	
10–40	0 (0)
50	5 (2.5)
60	16 (8.0)
70	68 (34.0)
80	86 (43.0)
90	25 (12.5)
100	0 (0)
Korean ESAS (median, range)	
Pain	3.0 (0.0–10.0)
Fatigue	4.0 (0.0–10.0)
Nausea	0.0 (0.0–9.0)
Depression	2.0 (0.0–8.0)
Anxiety	2.0 (0.0–9.0)
Drowsiness	3.0 (0.0–10.0)
Shortness of breath	2.0 (0.0–10.0)
Sleep disturbance	2.0 (0.0–10.0)
Loss of appetite	5.0 (0.0–10.0)
Peaceful	5.0 (0.0–10.0)
Total score	31.0 (1.0–81.0)
Median survival, months [range]	7.6 [0.2–36.8]

ESAS, Edmonton Symptom Assessment System; KPS, Karnofsky Performance Status; *n*, number.

### Accuracy of CPS, KPS, and ESAS score

**Fig 1.**
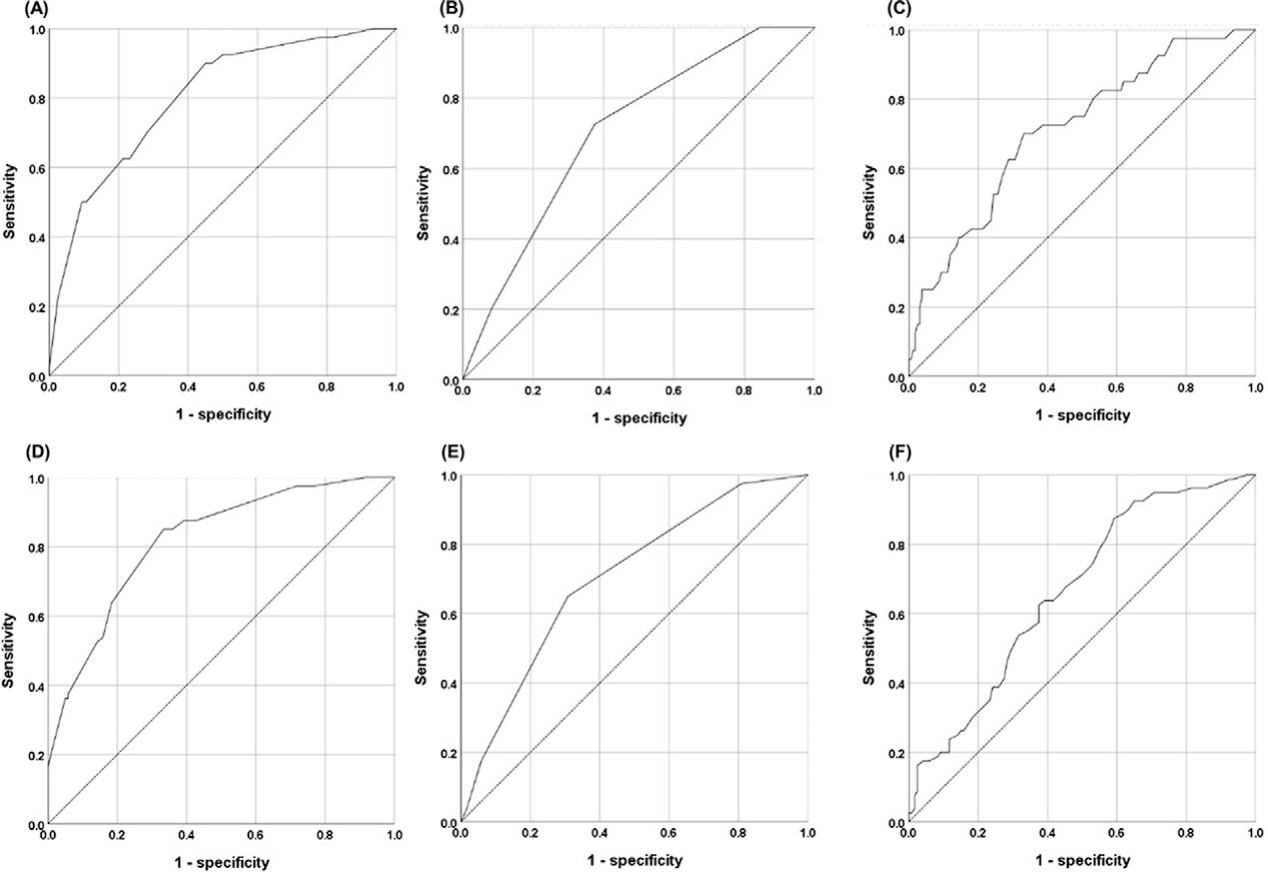
Receiver operating characteristic (ROC) curve for a 3-month prediction of **(A)** Clinician’s Prediction of Survival (CPS), **(B)** Karnofsky Performance Status (KPS), and **(C)** Edmonton Symptom Assessment System (ESAS) total score; for a 6-month prediction of **(D)** CPS, (E) KPS, and **(F)** ESAS total score.

**Fig 2.**
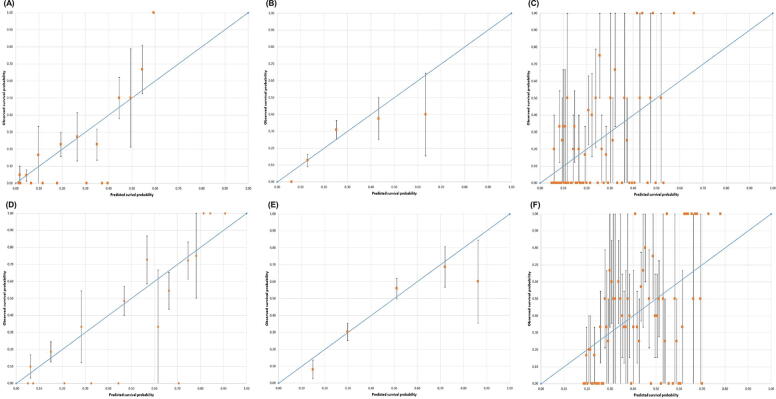
Calibration plot for 3-month prediction of **(A)** CPS, **(B)** KPS, and **(C)** ESAS total score; for 6-month prediction of **(D)** CPS, **(E)** KPS, and **(F)** ESAS total score.

Eight medical oncologists with a median of 14.0 years of experience evaluated the CPS. The patient characteristics are listed in Table 2. The sensitivity, specificity, PPV, NPV, and OA of prediction of the 3- and 6-month survival are shown in Table 3. Sensitivity of 6-month survival prediction was the highest in CPS (≤6 months). Specificities for 3-month survival prediction were more than 90% in both CPS (≤3 months) and KPS (≤60). In terms of predicting 3-month survival, the values of AUROC of CPS, KPS, and ESAS total score were 0.80 (95% CI: 0.73–0.88), 0.71 (95% CI: 0.62–0.79), and 0.71 (95% CI: 0.62–0.81), respectively. Regarding 6-month survival, the values of AUROC of CPS, KPS, and ESAS total score were 0.82 (95% CI: 0.76–0.88), 0.70 (95% CI: 0.63–0.78), and 0.63 (95% CI: 0.55–0.71), respectively. Details of the AUROC with 95% CI: are shown in Table 4 and Figure 1. Calibration plots demonstrated a correlation between the predicted and observed OS values (Fig. 2). Calibration plots of the CPS, KPS, and ESAS total score for 3-month and 6-month survival showed good overall concordance between the predicted and observed survival probabilities.

**Table 2.** Physician Characteristics

Characteristics	Medical oncologist (*n* = 8)
Age, years (median, range)	39 (33–45)
Female	4 (50%)^a^
Years of clinical experience, median (interquartile range)	14.0 (9.5–17.0)
Palliative care training	3 (37.5)

^a^ Descriptive data are presented as numbers (%).

**Table 3.** Prediction of the 3- and 6-Month Survival of CPS, KPS, and ESAS Score

	Prevalence, *n* (%)	Sensitivity (%)	Specificity (%)	PPV (%)	NPV (%)	OA (%)
3-month survival						
CPS ≤ 3 months	35 (17.5)	50.0	90.6	57.1	87.9	82.5
KPS ≤ 60	21 (10.5)	20.0	91.9	38.1	82.1	77.5
ESAS total score > 30	101 (50.5)	72.5	55.0	28.7	88.9	58.5
6-month survival						
CPS ≤ 6 months	108 (54.0)	85.0	66.7	63.0	87.0	74.0
KPS ≤ 60	21 (10.5)	17.5	94.2	66.7	63.1	63.5
ESAS total score > 30	101 (50.5)	63.8	58.3	50.5	70.7	60.5

ESAS total score: sum of 10 ESAS items.

CPS, Clinician’s Prediction of Survival; NPV, negative predictive value; OA, overall accuracy; PPV, positive predictive value.

**Table 4.** Areas Under Receiver Operating Curve (AUROCs) and 95% Confidence Interval of CPS, KPS, and ESAS Score

	3-month survival	6-month survival
CPS	0.80	0.82
	(0.73–0.88)	(0.76–0.88)
KPS	0.71	0.70
	(0.62–0.79)	(0.63–0.78)
ESAS total score	0.71	0.67
	(0.62–0.80)	(0.59–0.74)

ESAS total score: sum of 10 ESAS items.

## Discussion

The current study aimed to provide useful parameters for deciding a timely transition to palliative care. In our findings, the CPS proved to be the most accurate in patients with a survival time in terms of months. KPS was the second most accurate prognostic marker for predicting the 3-month and 6-month survival rates. Considering the difficulties in formulating the CPS, KPS might help the inexperienced physicians to predict survival.

Prognostic information is essential, being a priority for patients with advanced cancer, focusing on an individual’s end-of-life care goal. Performance status, represented by KPS, has been demonstrated as a strong single factor influencing survival outcomes in advanced cancer.^33,34^ CPS is a simple and commonly used tool for predicting survival.^35,36^ However, CPS tends to be optimistic and can be influenced by physicians’ experience and competence. Furthermore, the CPS is flexible enough to be used at any stage of illness. CPS is particularly helpful when test results or laboratory findings are unavailable.^27,35,37^ Notably, CPS has been reported to be very accurate in predicting the last weeks of survival.^26,37^ A recent clinical practice guideline suggested that the CPS could serve as a standard prognostic evaluation for patients with cancer in the last months of life.^23^ More than 90% specificity was identified in predicting both the 3-month survival by CPS and the 3-month and 6-month survival by KPS. Higher specificities of both CPS and KPS could be helpful in predicting death within 3 months. CPS showed the highest sensitivity for predicting the 6-month survival. Prognostic information is an important component of a successful ACP.^38,39^ Recent studies in Asia have suggested that patients prefer communication with their physicians regarding ACP, based on their prognostic awareness.^40,41^ Therefore, CPS may enhance physicians’ competency in facilitating ACP conversations with patients and families. For instance, experienced physicians can use CPS as a screening tool in advanced care planning. It is necessary for physicians to acquire substantial clinical experience in formulating CPS without hesitation. However, inexperienced clinicians may face challenges when formulating CPS. In such cases, KPS aids inexperienced physicians in making clinical decisions regarding palliative care.

In our study, CPS, KPS, and the sum of ESAS demonstrated acceptable AUROCs (≥0.7) in predicting survival at 3 months. In particular, CPS was proven to be superior to other tools, with an AUROC >0.80. Despite the scarcity of data on predicting the survival of patients, whose survival is intermediate between 3 and 6 months, previous research reported that the AUROC of CPS is 0.62–0.65.^28,42^ A possible explanation for the higher CPS in our study could be physician related. Medical oncologists, who evaluate the CPS, are also in continuous charge of palliative care; thus, they may be more befitting for accurate prediction than those reported in previous studies.

Patient-reported outcome (PRO) is known to predict survival in patients with advanced cancer, because it reflects cancer-related symptom burdens, such as anorexia and dyspnea.^20^ Notably, the ESAS is commonly used to screen and monitor patient symptoms in all disease trajectories, including palliative settings. Previous studies had demonstrated that ESAS is an independent prognostic tool for predicting survival prediction.^19,43,44^ Subbiah et al. suggested that a high ESAS global distress score is associated with overall survival of patients with cancer and could be a sign of impending death.^20^ Our findings showed that the ESAS total score is useful for predicting 3-month survival. Sensitivity of the ESAS total score for predicting 3-month survival was 72.5%. This sensitivity was the highest among the prognostic tools for predicting 3-month survival. Thus, total ESAS can be used to screen for palliative referral before it is delayed. In the near future, if valid electronic tools are introduced, PRO will help busy clinicians obtain important prognostic information from the patients’ perspectives.

Our study had some limitations. First, it was conducted at a single tertiary cancer center in South Korea. Thus, the results may not be generalizable to other care settings. Second, patients were enrolled based on physicians’ prediction of 1-year survival. Thus, selection bias of participants may have occurred due to the overestimation of CPS. Third, many years have passed since initial patient enrollment and reporting of the study results. We recognize that our results may not fully reflect the possible influences of innovative treatments, such as immunotherapy and currently available antibody–drug conjugates. Finally, the patients’ overall survival could be influenced by population density and medical service levels. We did not collect information regarding the place of death, which might be another limitation. However, we assumed that approximately one-third of the patients died at the study hospital, and approximately two-thirds of the patients died at inpatient hospices in other hospitals.^45^ A remarkable strength of our study is that we included more than sixty-five percent of the patients undergoing chemotherapy. Previous studies had not included a sufficient number of patients undergoing chemotherapy; thus, we believe that our results would be easily applicable to those populations. Our data proposed the performance of CPS for 6-month survival and that of ESAS total score for 3-month survival as screening tools for patients with advanced cancer. There is still a paucity of studies on patients with survival in months. Survival duration is known to be intermediate, and CPS is known to be inaccurate during this period.^45^ Our findings indicated that CPS has the greatest potential to facilitate timely palliative care. However, CPS can depend on a physician’s experience and confidence^46^; therefore, inexperienced clinicians may use the KPS to complement their CPS in clinical settings, and further research on patients in diverse settings is warranted.

In conclusion, we compared the accuracy of various prognostic tools in patients using monthly survival rates. CPS was considered the most accurate tool for initiating timely palliative transition in patients with monthly survival. The sum of the ESAS variables can also be considered a screening tool to discuss end-of-life care before the appearance of refractory symptoms or unconsciousness. We suggested KPS to be used instead of CPS to predict intermediate survival.

## Abbreviations Used

ACP: advanced care planning
AUROC: area under the receiver operating characteristic curve
CPS: clinicians’ prediction of survival
ESAS: Edmonton Symptom Assessment System
KPS: Karnofsky Performance Scale
NPV: negative predictive value
OA: overall accuracy
OS: overall survival
PPV: positive predictive value
PRO: patient-reported outcome
QoL: quality of life
