# Supplementary material for: Clinicians’ Prediction of Survival Is Most Useful for Palliative Care Referral
Source: Palliat Med Rep. 2024 Aug 21;5(1):365–72. doi: 10.1089/pmr.2024.0013 (PMC11392678; doi:10.1089/pmr.2024.0013)
Supplement: Supplementary Table S1 [file pmr.2024.0013_supplementary_data.pdf]

Supplementary Table 1. Details of Chemotherapy Performed at Enrollment (n = 131)

Variable	n (%)
**Primary cancer site**	
Lung	50 (38.2)
Stomach	8 (6.1)
Colon/Rectum	11 (8.4)
Ovary/Cervical	3 (2.3)
Esophagus	5 (3.8)
Head/Neck	1 (0.8)
Soft/tissue	6 (4.6)
Kidney/Bladder	23 (17.6)
Breast	17 (13.0)
Others	7 (5.3)
**Number of received chemotherapy**	
1	25 (19.1)
2	40 (30.5)
≥ 3	66 (50.4)
**Treatment at enrollment**	
Cytotoxic chemotherapy	109 (83.2)
Tyrosine kinase inhibitor	12 (9.2)
Immunotherapy	9 (6.9)
Others	1 (0.8)
**Previous treatment history**	
Cytotoxic chemotherapy	127 (96.9)
Tyrosine kinase inhibitor	29 (22.1)
Immunotherapy	23 (17.6)
Others	13 (9.9)
